# The hydrocephalus inducing gene product, Hydin, positions axonemal central pair microtubules

**DOI:** 10.1186/1741-7007-5-33

**Published:** 2007-08-07

**Authors:** Helen R Dawe, Michael K Shaw, Helen Farr, Keith Gull

**Affiliations:** 1 Sir William Dunn School of Pathology, University of Oxford, South Parks Road, Oxford, OX1 3RE, UK

## Abstract

**Background:**

Impairment of cilia and flagella function underlies a growing number of human genetic diseases. Mutations in *hydin *in *hy3 *mice cause lethal communicating hydrocephalus with early onset. Hydin was recently identified as an axonemal protein; however, its function is as yet unknown.

**Results:**

Here we use RNAi in *Trypanosoma brucei *to address this issue and demonstrate that loss of Hydin causes slow growth and a loss of cell motility. We show that two separate defects in newly-formed flagellar central pair microtubules underlie the loss of cell motility. At early time-points after RNAi induction, the central pair becomes mispositioned, while at later time points the central pair is lost. While the basal body is unaffected, both defects originate at the basal plate, reflecting a role for TbHydin throughout the length of the central pair.

**Conclusion:**

Our data provide the first evidence of Hydin's role within the trypanosome axoneme, and reveal central pair anomalies and thus impairment of ependymal ciliary motility as the likely cause of the hydrocephalus observed in the *hy3 *mouse.

## Background

Hydrocephalus is the accumulation of cerebrospinal fluid within the brain ventricles, which enlarge to pathological levels in the presence of excessive cerebrospinal fluid. The ventricles are covered with a ciliated epithelium called the ependyma, and dysfunctional cilia can be associated with hydrocephalus [1-5]. Mutations in the hydrocephalus-inducing gene, *hydin*, in *hy3 *mice cause lethal communicating hydrocephalus with early onset [6,7] and the corresponding region within human chromosome 16 is also associated with congenital hydrocephalus [8].

Cilia and flagella are highly conserved structures typically made up of an axoneme consisting of nine outer doublet microtubules. In addition, motile cilia generally possess inner and outer dynein arms on the doublet microtubules, radial spokes, and a central pair of two singlet microtubules. Hydin is an axonemal protein. The first evidence for this came from localisation of the *hydin *transcript to ciliated ependyma in addition to spermocytes and oviduct and respiratory epithelia [6]. Further support for this idea comes from the recent completion of proteomic studies of eukaryotic cilia and flagella from *Trypanosoma, Chlamydomonas *and *Tetrahymena *[9-11], which enabled us to conduct a direct comparison of axonemal components from three diverse eukaryotes. Intriguingly, Hydin was one of very few proteins to be identified in all three proteomes [9]. The presence of Hydin in three diverse ciliary/flagellar proteomes demonstrates definitively that it is an axonemal protein: it cannot be solely a basal body protein as neither the *Chlamydomonas *or *Tetrahymena *preparations included basal bodies. Neither can it be a component of the kinetoplastid-specific paraflagellar rod, which was present in the trypanosome flagellar preparation. Hydin is most likely a structural protein as it is found within the axonemal fraction of the *Chlamydomonas *flagellar proteome [10] and trypanosome Hydin is resistant to extraction by detergent and 1 M salt [9]. The absence of a Hydin homologue from the *Caenorhabditis elegans *genome is suggestive of a role in ciliary/flagellar motility, as *C. elegans *builds only immotile sensory cilia. Furthermore, we also showed that RNA-interference (RNAi) mediated ablation of the trypanosome Hydin orthologue led to a motility defect consistent with a flagellar role [9]. However, Hydin's function within cilia and flagella remains unknown.

The protozoan parasite *Trypanosoma brucei *is an excellent model organism in which to study cilia and flagella due to its well characterised cytoskeleton (for a review, see [12]) and its genetic tractability. It possesses a single flagellum, which exits at the posterior end of the cell from the flagellar pocket and is attached to the cell body along its length, proceeding to the anterior tip of the cell. As well as the axoneme, the flagellum contains an additional structure, the paraflagellar rod (PFR), which runs alongside the axoneme, to which it is attached. Unusually, at cell division the cell maintains the old flagellum and assembles a new flagellum for the daughter cell.

Here, we use the tractable reverse genetics of *T. brucei *to address the function of Hydin. We show that two separate defects in central pair microtubules underlie the loss of flagellar motility, revealing central pair anomalies and thus impairment of ependymal ciliary motility as the likely cause of the aetiology of this disease.

## Results

### Ablation of TbHydin causes slow growth and motility defects

To provide a functional explanation for the hydrocephalus observed in *hy3 *mice, we cloned a 580 base pair fragment of the 5' end of the *TbHydin *gene (GeneDB accession number Tb927.6.3150) and used inducible RNAi in the single-celled model organism *T. brucei *to ablate protein expression. RNAi in trypanosomes is distinct from the siRNA commonly used in mammalian cell systems in that it involves the *in vivo *production of multiple non-overlapping short-interfering RNAs by the endogenous RNAi machinery in the trypanosome. If we assume an average length of 21 nucleotides for most short interfering RNAs (as used in mammalian cells), the 580 bp starting region gives at least 27 non-overlapping knockdown constructs that will likely be operating in this RNAi situation.

Quantitative real-time PCR was used to measure the decrease of Hydin transcript levels following RNAi induction; total transcript levels at 72 h after RNAi induction were 11% of the parental (29–13) cell line. The specificity of the TbHydin RNAi was assessed using inducible RNAi against two further trypanosome proteins: PACRGA and PACRGB, which are components of the trypanosome flagellum [9,13] that give no phenotype (assessed by growth, motility, organelle position and flagellar ultrastructure, [13]; see also Additional file 1) on RNAi, despite efficient RNA depletion [13]. Thus, the flagellar ultrastructural phenotype that we report in this work is unlikely to be attributable to unexpected non-specific trypanosome RNAi effects.

**Figure 1:**
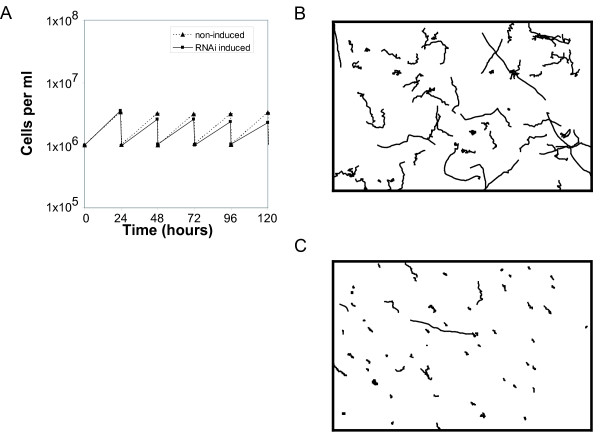
**TbHydin RNAi-induced cells grow slowly and do not locomote**. (A) Representative growth curve shows growth defect starting 48 h after RNAi induction in TbHydin RNAi-induced cells (black squares) compared to non-induced controls (triangles). Cells were maintained in log phase by diluting the culture every 24 h. (B and C) Trajectories of individual cells over the course of a 40 s time-lapse. While non-induced control cells (B) are motile, cells locomotion is severely compromised in TbHydin RNA-induced cells (C).

RNAi against TbHydin resulted in a progressive slow growth phenotype first apparent at 48 h post-induction (Figure 1A). Analysis of nuclear and kinetoplast (mitochondrial DNA) content revealed a small defect apparent from 72 h after RNAi induction: 3% of the population contained more than two nuclei, suggestive of a minor defect in cytokinesis. No evidence of the cell clumping or aggregation as sometimes observed in other flagellar mutants [14,15] could be observed until over 5 days after induction. However, more detailed examination revealed that TbHydin RNAi-induced cells had difficulties in segregating their kinetoplasts/basal bodies (data not shown) and this is probably sufficient to explain the growth defect, as impairment of cell morphogenesis tends to be lethal in trypanosomes. TbHydin RNAi-induced cells possessed flagella of normal length (20.2 μm ± 0.5 in non-induced cells (n = 100) compared with 20.3 μm ± 0.6 in induced cells (n = 100)) and continued to build a new flagellum each cell cycle. However, cell motility was strongly affected. We first observed a loss of cell motility at 48 h after induction [9], concomitant with the appearance of the growth defect, suggesting that the two are linked and that flagellar motility is required for kinetoplast segregation. By tracking locomotion of a cohort of individual cells in the population 72 h after induction of RNAi over the course of 40 s we were able to assess cell locomotion. While non-induced control cells were motile and locomoted over the course of the time-lapse (Figure 1B, n = 59), there was a significant decrease in the locomotion of TbHydin RNAi-induced cells (Figure 1C, n = 61, p < 0.001, Mann-Whitney test) with the majority of TbHydin RNAi-induced cells completely failing to locomote (Figure 1C).

### TbHydin is required for central pair microtubule positioning

To investigate the reason for the flagellar motility defect, we prepared TbHydin RNAi-induced cells for ultrastructural examination by transmission electron microscopy. We observed significant differences in the arrangement of the 9+2 axoneme, with an early effect on central pair positioning first apparent 48 h after induction, and a later effect on central pair nucleation visible from 72 h post-induction.

**Figure 2:**
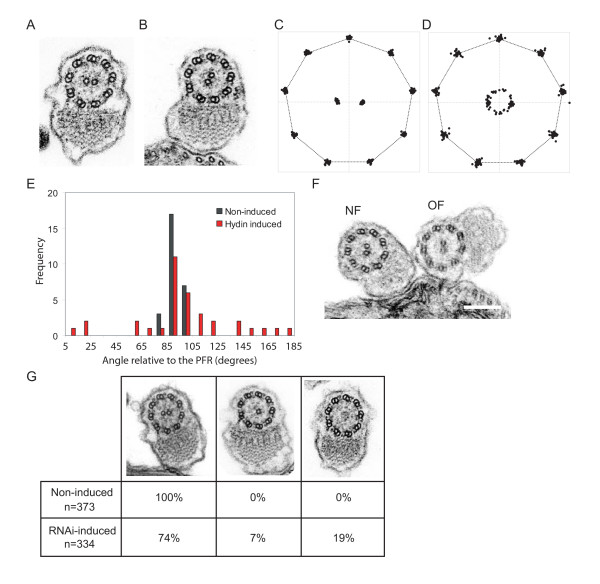
**Central pair microtubule defects in TbHydin RNA-induced cells**. (A and B) Transmission electron microscopy images of TbHydin non-induced cells (A) and RNAi-induced cells 48 h after induction (B). Note the aberrant position of the central pair microtubules in (B) compared to the non-induced control. (C-E) Graphical representation of the central pair mispositioning in TbHydin RNAi-induced flagellar profiles (D and red bars in E) compared to the fixed central pair position seen in non-induced controls (C and black bars in E). (F) A cell late on in the cell cycle with two flagella. The new flagellum (NF) has a misoriented central pair while the old flagellum (OF) is normal. (G) Loss of the central pair microtubules in TbHydin RNA-induced cells. Flagellar profiles were analysed at 72 h after RNAi induction, and loss of one or both of the central pair microtubules quantified. Scale bars = 200 nm.

In *T. brucei*, the extra-axonemal PFR is attached to the axoneme via connections to outer doublets 4 and 7 and thus provides a reference point for the position of the central pair microtubules. In kinetoplastids including *T. brucei*, the two central pair microtubules have a fixed orientation parallel to the extra-axonemal PFR and do not rotate during flagellar beating [16]. TbHydin non-induced control cells maintained this positioning (Figure 2A), however around 50% of TbHydin RNAi-induced cells exhibited loss of the fixed central pair position (Figure 2B). We used a geometric correction [16] to transform the elliptical cross-sections obtained from the microscope to circular ones representative of true transverse sections and examined the degree of central pair misalignment. We found that in non-induced cells the axonemal profiles displayed almost perfect ninefold symmetry and the angle of the central pair relative to the PFR was very consistent (Figure 2C, E, black bars, n = 30). In the absence of TbHydin, the central pair was variably distributed around the axis in approximately 50% of axonemal profiles examined (Figure 2D, E, red bars, n = 35). The angle of the central pair relative to the PFR is 92.7 ± 0.5 in *T. brucei *[16]. While only 11% of non-induced sections varied from this by more than 5%, 71% of TbHydin RNAi-induced sections showed greater than 5% variation from this figure, with an average angle relative to the PFR of 98.1 ± 38.3. The remainder had an apparently wild-type orientation, although as there was no easy way to distinguish the two central pair microtubules we cannot discount the possibility that a 180° misorientation had occurred in a proportion of these. In addition, given that we expect only new flagella to be affected by the RNAi, some of these will obviously represent old flagella built before RNAi induction.

As RNAi targets RNA and not protein, flagellar proteins present in flagella built before RNAi induction remain present and disappear only on turnover. A protein can therefore be missing in the newly built flagellum but still be present in the old flagellum [17,18]. We observed cells early on in induction (48 h after addition of doxycycline) that were in division and had two flagella (the old flagellum and the new flagellum). Late in the cell cycle it is possible to distinguish between the two with ease: the new flagellum is always present on the left-hand side of the old flagellum when viewed from the posterior end of the cell [19]. Of the cell profiles examined, 18% had two affected flagella, 37% had an affected new flagellum and an unaffected old flagellum (Figure 2E), while the remainder had two unaffected flagella. In no case did we observe an affected old flagellum without an affected new flagellum, suggesting Hydin is needed for axoneme formation rather than maintenance.

At 72 h after RNAi induction an additional defect, loss of central pair microtubules, could be seen with several axonemal profiles lacking both central pair tubules (9+0 configuration), and a smaller number lacking one central pair microtubule (9+1 configuration), similar to the phenotype observed in other trypanosome flagellar RNAi mutants [14,16]. We quantified this effect by analysing axonemal profiles from both non-induced (n = 373) and TbHydin RNAi-induced (n = 334) preparations (Figure 2F). While all the non-induced profiles displayed a canonical 9+2 axonemal configuration, 19% of TbHydin RNAi-induced cells exhibited a 9+0 configuration and a further 7% had a 9+1 configuration. The remainder had a 9+2 configuration, with many exhibiting the expected misorientation of the central pair. In the case of the 9+1 axonemes, we have no evidence to suggest a preference for loss of either tubule. In *T. brucei *there is as yet no easily observable distinction between the two microtubules. Moreover, although the single remaining central tubule did not collapse in to the centre of the axoneme, its fixed orientation was lost (data not shown), precluding its identification by positioning alone.

### The central pair defects originate at the basal plate

**Figure 3:**
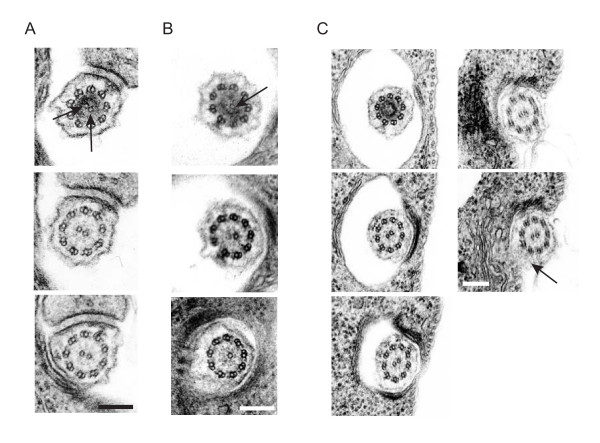
**Central pair defects originate at the basal plate**. (A-C) Serial thin section electron micrographs through the basal plate region of non-induced (A) and TbHydin RNAi-induced (B and C) cells. In non-induced cells, the two central pair tubules are nucleated simultaneously (A, arrows), however only a single central pair tubule is present in the TbHydin RNA-induced cell (B, arrow). (C) serial thin sectioning of TbHydin RNA-induced cells from the basal plate through to where the flagellum exits the flagellar pocket demonstrates that the central pair mispositioning defect also originates at the basal plate. Central pair position is determined relative to the PFR (arrow). Note how this position is invariant throughout the series. Scale bars = 200 nm.

These data provide direct evidence that Hydin functions to position and stabilise central pair microtubules. We examined the ultrastructure of the basal body and transition zone to determine if the defects originated at these points or more distally along the flagellum. The *T. brucei *basal body is a canonical nine-triplet microtubule structure that extends into a transition zone made up of nine doublet microtubules lacking the central pair. The central pair is nucleated at an electron-dense site called the basal plate, which is the location of the microtubule nucleating factor gamma tubulin [20,21], which specifically acts to nucleate the central pair microtubules during axoneme morphogenesis [18]. Each of these structures was present in TbHydin RNAi-induced cells and appeared indistinguishable to non-induced cells (data not shown). Serial thin sectioning through the basal plate region revealed that in control cells both central pair microtubules are nucleated simultaneously (Figure 3A, arrows point to the newly forming microtubules within the basal plate). By contrast, when we examined TbHydin RNAi-induced cells with a 9+1 axonemal configuration within the flagellar pocket, we found that only a single microtubule was present at the basal plate (Figure 3B, arrow), suggesting that loss of TbHydin either blocks central pair assembly or destabilises the central pair so much that it completely collapses.

To determine if the defect in central pair positioning also originates at the basal plate, we performed serial sectioning through the flagellar pocket from the basal plate region to the point outside the flagellar pocket where the PFR is first apparent (Figure 3C, the arrow in the final image indicates the forming PFR structure). This covers a region of 1.2–1.5 μm; five sections from a single representative series are shown here. The angle of the central pair is defined by its position relative to the PFR in the last image. It is clear that the central pair is misaligned and that this originates at the site of nucleation as the angle of the central pair is invariant throughout the series, again suggesting that the central pair defects originate at the basal plate region.

## Discussion

Hydrocephalus is a common birth defect, with an estimated incidence of 1 in 1000 live births [22]. It can have many causes: impaired cerebrospinal fluid flow, excess fluid production or impaired fluid absorption and can be congenital or acquired. There is no cure and the usual treatment is the surgical insertion of a ventricular shunt to drain the excess cerebrospinal fluid and relieve intracranial pressure, a procedure often complicated by infections and malfunctions. A better understanding of the molecular mechanisms of the disease is therefore vital for the development of improved treatments.

Cerebrospinal fluid (CSF) is continuously produced within the brain ventricles and drains into the subarachnoid spaces from where it is reabsorbed. The choroid plexus produces most of the CSF, the rest being produced by extracellular fluid flow from the brain parenchyma through the ependymal cells [23]. The mechanism of hydrocephalus formation in Hydin mutants remains unknown. Hydrocephalus in the *hy3 *model was originally suggested to be caused by defective CSF reabsorption [24,25] as dye injection experiments in *hy3 *homozygotes demonstrated that fluid flow between the ventricles could occur, but dye injected into the ventricles did not enter the subarachnoid space. However, the mice examined were already late on in the progression of hydrocephalus and the localisation of *hydin *to ependymal cells [6] makes it unlikely that this is the complete picture. The phenotypic analysis of *hy3 *mice prior to the onset of hydrocephalus will be necessary to distinguish the initiating event for hydrocephalus development from secondary effects, as defective CSF absorption can occur later.

Hydin is not required for cilium formation, as our data reveal that trypanosome Hydin mutants still form a flagellum and *hy3 *mutant mice form cilia on the ependyma and choroid plexus [26]. Within the brain, *hydin *localises to ependymal cells [6]; hence our data implicate a loss of central pair function within ependymal cilia in hydrocephalus development. The central pair microtubules are critical for the motility of most cilia and flagella, and mutations that affect the central pair have severe consequences for axonemal beating [5,14,15,27-30]. The presence of the central pair microtubules is absolutely required for motile cilia function within the brain: mice deficient in the central apparatus protein SPAG6 [5] lack the central pair microtubules and develop severe hydrocephalus. Males that survive to adulthood are infertile with abnormalities in sperm structure and motility, indicating that SPAG6 is also required to maintain the structural integrity of sperm. *Hydin *is also found in developing spermatocytes and ciliated epithelia lining the oviduct and bronchi [6]. As the hydrocephalus is always lethal before the mice reach maturity [31] it is unknown whether Hydin plays similar roles in these tissues, although male infertility has been reported [31].

As Hydin is required for ciliary motility, the initiating factor for hydrocephalus could be impaired CSF flow due to reduced ciliary beat, similar to that observed in mice deficient in the outer dynein arm protein Dnahc5 [4]. An alternative possibility is that ciliary malfunction could lead to alterations in the ependymal layer and changes to CSF production. The pleiotropic phenotype of the Tg737^*orpk *^mutant mouse [32-36], which lacks the cilia assembly protein IFT-88/Polaris [34,37], includes hydrocephalus caused by alterations in the ion transport activity of the ependymal cells and consequent overproduction of CSF [1]. Indeed, *hy3 *mice have an increase in lipid in cells of the choroid plexus and ependyma [26], suggesting that Hydin function might be needed for cell homeostasis and/or signalling.

Bioinformatics yields few additional clues as to Hydin's function: a novel N-terminal domain distantly related to the major sperm protein domain was recently identified and named ASH for its presence in the microcephaly-associated protein ASPM, SPD-2 and Hydin [38]. Evidence from other proteins containing the domain suggests that ASH domain proteins play important roles in cilia or centrosomes and might possess microtubule binding functions [38]. The Hydin sequence also contains an adenylate kinase motif similar to that found in the *Chlamydomonas *central pair protein Cpc1 [38]. Whether Hydin can catalyse ATP generation within cilia and flagella remains to be seen; it should be noted, however, that the adenylate kinase domain within Cpc1 appears to be inactive and instead acts as a linker protein that attaches enolase to the central pair [39].

Our data suggest that one function of TbHydin is to regulate the position of the central pair at the basal plate. The presence of the basal plate in TbHydin RNAi-induced cells makes it unlikely that Hydin is the sole protein that makes up this region although it might be a component. However, the presence of Hydin homologues in the *Chlamydomonas *[10] and *Tetrahymena *[11] flagella/cilia proteomes argues against Hydin function being restricted to the basal plate as material for these studies were prepared by deflagellation, a process which leaves the basal plate within the cell body. It therefore seems most likely that Hydin is a component of the central pair itself. This idea is supported by Hydin's evolutionary distribution: while the *hydin *gene is conserved amongst many flagellate organisms, it is notably absent from the genome of *C. elegans*, which lacks motile cilia and makes only 9+0 axonemes lacking the central pair.

Misorientation of the central pair also occurs in mutant trypanosomes lacking the central pair proteins (PF16 and PF20, [14,15]), basal body proteins (delta tubulin and gamma tubulin, [16]) and dynein proteins (DNAI1, [14]). All these mutations result in a loss of flagellar motility, however the position of the central pair is not determined by flagellar beating as loss of the PFR protein PFR2 causes flagellar paralysis [40] but the central pair remains fixed in position [16]. Our data suggest that central pair orientation is determined at the basal plate and subsequently perpetuated along the length of the axoneme. Whether this orientation is constrained by interactions with other structures along the length of the axoneme remains to be seen. It should be noted, however, that severe disruption of the outer doublet microtubules caused by simultaneous depletion of the axonemal proteins PACRGA and B can also lead to a loss of correct central pair orientation [16] even in the presence of a native basal body configuration [13], suggesting that some constraint along the axoneme might occur.

It has been proposed that the central pair signals through the radial spokes to provide an asymmetric stimulus of the dynein motors associated with each of the outer doublet microtubules that enables a flagellar bend to be generated. Indeed, asymmetric contacts between radial spokes and the central pair have been observed in molluscan gill cilia [41], indicating that the central pair might transiently interact with different radial spokes during ciliary beating. One hypothesis for how this asymmetric stimulus is generated relies on the rotation of the central pair microtubules observed in *Chlamydomonas *[42] and *Paramecium *[43,44], with rotation of an asymmetric central pair regulating the successive activation and deactivation of dyneins around the axoneme. However, central pair rotation is not found in all organisms. Ctenophore comb plate cilia, echinoderm sperm flagella, and flagella of various kinetoplastida all exhibit fixed central pair orientation [16,45,46], raising the question of how the central pair regulates motility in these systems. Our data confirm previous studies [16] that the central pair does not rotate in trypanosomes but we cannot yet say whether the misoriented central pair that we observe in TbHydin mutants is the cause of the motility phenotype observed here, or a consequence of it. Determining the orientation of the central pair in other organisms is made difficult by the lack of an extra-axonemal reference point, hence it remains unknown whether the ependymal cilia of mammalian brain utilise central pair rotation during beating.

Organisms where the central pair rotates during axonemal beating, such as *Chlamydomonas*, have different phenotypes in mutants that affect the central pair to those organisms where the central pair is constrained. The central pair proteins PF20 and PF16 are essential for central pair assembly in *Chlamydomonas *[27,28,47,48], while misorientation is the major defect observed in trypanosomes [14,15] and similar to the data described here, a proportion of the cells also show loss of one or both central pair microtubules [14]. The central-pair microtubules are also destabilised in sperm flagella of PF16 and PF20 mouse knock-out mutants [5,49]. However, as there is no extra axonemal-reference point comparable to the PFR in mice, it remains unknown whether the central pair positioning is also affected in these mutants. The issue of organism to organism comparison of phenotype is, however, not straightforward as the type of mutation often varies according to the genetic tractability of each system. Hence, a complexity arises in defining mutant phenotypes derived via knockout, RNAi depletion, point mutation, insertion etc, highlighting the need for caution in direct comparisons.

## Conclusion

Many putative ciliary disease genes have been identified over the last five years. Many of these are highly evolutionarily conserved proteins for which the biological function is unknown. Our work identifies TbHydin as a protein that plays a role in axonemal stability by ensuring correct positioning of the central pair microtubules and reveals central pair dysfunction as the likely cause of the hydrocephalus observed in the *hy3 *mouse. A challenge for the future will be to identify the axonemal compartment where trypanosome Hydin is located and to elucidate the molecular mechanisms that underlie its function.

## Methods

### Constructs and trypanosome transfection

PCR was used to amplify a 580 base pair fragment of the 5' end of the TbHydin gene (GeneDB accession number Tb927.6.3150) using the following specific primers: AAGGGTAACGAGGGAACG (TbHydin forward) and GACGCCGGAAGAAGGAGA (TbHydin reverse) incorporating *Xba*I and *Hin*dIII restriction sites into the forward and reverse primers, respectively. Fragments were cloned into the p2T7-177 inducible RNAi vector [50] using the *Xba*I and *Hin*dIII sites. Primers and constructs for PACRGA and PACRGB were as published [13]. Procyclic *T. brucei *29–13 [51] cells were transfected using standard protocols, selected using 5 μg/ml phleomycin, and cloned by limiting dilution. Quantitative real-time PCR was used to measure the decrease of Hydin transcript levels following RNAi induction, as described previously [13]. Total transcript levels at 72 h after RNAi induction were 11% of the parental (29–13) cell line.

### Trypanosome culture

Cell lines were cultured in SDM 79 medium [52] supplemented with 10% foetal calf serum and appropriate antibiotics at 28°C. For RNAi, cells were induced using 1 μg/ml doxycycline.

### Cell growth and motility analysis

Cell growth was monitored using a haemocytometer and graphs were constructed using the average from two independent counts. For motility analyses, non-induced cells and induced cells after 72 h were grown to a density of 2 × 10^6^ cells/ml. The motility of these cells was analysed as described [53]. Cells were tracked for 20 frames, taken over 40 s and the position of each cell tracked over time manually using NIH Image.

### Analysis of nucleus and kinetoplast number

Cells were settled onto glass slides, extracted in 1% NP-40 in 100 mM PIPES, pH 6.9, 2 mM EGTA, 1 mM MgSO_4_, 0.1 mM EDTA to yield cell cytoskeletons, fixed in 3.6% formaldehyde (TAAB) in phosphate buffered saline (PBS) and embedded in Vectashield (Vector Laboratories, Burlingham, CA) with 4,6-diamidino-2-phenylindole (DAPI). Slides were examined on a Zeiss Axioplan 2 microscope (Carl Zeiss Ltd, Welwyn Garden City, UK) using a 100 ×, 1.4NA oil immersion lens and the number of nuclei and kinetoplasts scored.

### Preparation of cells for thin-section TEM

Cells were fixed at 48 or 72 h after induction of RNAi in 2.5% gluteraldehyde, 2% paraformaldehyde and 0.1% picric acid in 100 mM phosphate (pH 6.5) for 2 h at 4°C followed by post-fixation in 1% osmium tetraoxide in 100 mM phosphate buffer (pH 6.5) for 1 h at 4°C. The fixed material was stained en bloc with 2% aqueous uranyl acetate for 2 h at 4°C. Following dehydration through a graded series of acetone and propylene oxide, the material was embedded in epon resin for sectioning.

### Statistical analysis

Statistical analyses were carried out using Excel (Microsoft Corp., Redmond, WA, USA).

## Note added in proof

During the review process the *Chlamydomonas reinhardtii *orthologue of Hydin was published and shown to be a central pair protein with a role in flagellar motility (see [54]).

## Authors' contributions

HRD made the TbHydin mutant, carried out the motility studies and electron microscopy, and drafted the manuscript. MKS participated in the electron microscopy. KG participated in the design and interpretation of the study and helped draft the manuscript. HF provided the EM negatives of PACRGA axonemes.

## Supplementary Material

Additional file 1 **RNAi of other trypanosome flagellar proteins does not affect the central pair**. Transmission electron microscopy images showing correct positioning of the central pair microtubules on inducible RNAi of the flagellar proteins PACRGA and PACRGB, as in the control 29–13 parental cell line, despite efficient RNA depletion [13]. Scale bar = 200 nm. Click here for file
