# Supplementary figures and images for: The hydrocephalus inducing gene product, Hydin, positions axonemal central pair microtubules
Source: BMC Biol. 2007 Aug 7;5:33. doi: 10.1186/1741-7007-5-33 (PMC2048497; doi:10.1186/1741-7007-5-33)

29-13 parental cell line

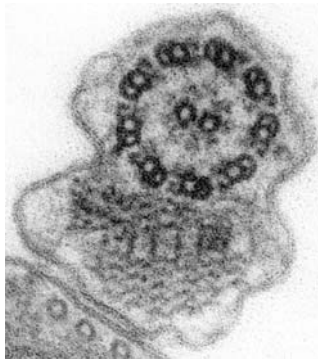

PACRGA RNAi-induced

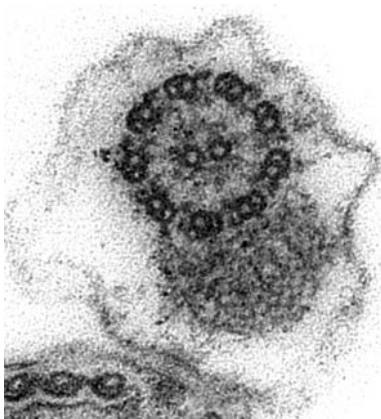

PACRGB RNAi-induced

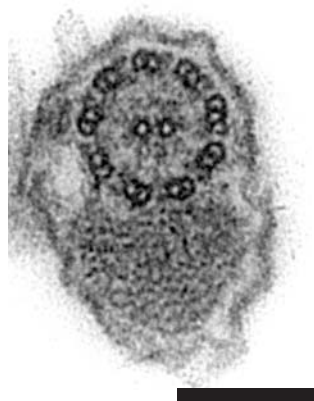

Supplement: Additional file 1 — RNAi of other trypanosome flagellar proteins does not affect the central pair. Transmission electron microscopy images showing correct positioning of the central pair microtubules on inducible RNAi of the flagellar proteins PACRGA and PACRGB, as in the control 29–13 parental cell line, despite efficient RNA depletion [13]. Scale bar = 200 nm. [file 1741-7007-5-33-S1.pdf]
